# *Inselect*: Automating the Digitization of Natural History Collections

**DOI:** 10.1371/journal.pone.0143402

**Published:** 2015-11-23

**Authors:** Lawrence N. Hudson, Vladimir Blagoderov, Alice Heaton, Pieter Holtzhausen, Laurence Livermore, Benjamin W. Price, Stéfan van der Walt, Vincent S. Smith

**Affiliations:** 1 Department of Life Sciences, Natural History Museum, Cromwell Road, London, SW7 5BD, United Kingdom; 2 Division of Applied Mathematics, Stellenbosch University, Stellenbosch 7600, South Africa; 3 Berkeley Institute for Data Science, University of California, Berkeley, CA, United States of America; University of Florida, UNITED STATES

## Abstract

The world’s natural history collections constitute an enormous evidence base for scientific research on the natural world. To facilitate these studies and improve access to collections, many organisations are embarking on major programmes of digitization. This requires automated approaches to mass-digitization that support rapid imaging of specimens and associated data capture, in order to process the tens of millions of specimens common to most natural history collections. In this paper we present *Inselect*—a modular, easy-to-use, cross-platform suite of open-source software tools that supports the semi-automated processing of specimen images generated by natural history digitization programmes. The software is made up of a Windows, Mac OS X, and Linux desktop application, together with command-line tools that are designed for unattended operation on batches of images. Blending image visualisation algorithms that automatically recognise specimens together with workflows to support post-processing tasks such as barcode reading, label transcription and metadata capture, *Inselect* fills a critical gap to increase the rate of specimen digitization.

## Introduction

There are an estimated two billion specimens stored in natural history collections worldwide [1]. These botanical, zoological, anthropological, geological, mineralogical, and paleontological collections represent the largest and most significant part of the available scientific evidence base of the planet’s biosphere. Collectively these specimens form a global research infrastructure for tackling major scientific challenges such as environmental change, biodiversity loss, human health, sustainable agriculture, and the exploration of scarce minerals [2–5]. Museum specimens have been used to estimate the regional species richness of tropical insects [6], to develop novel species-distribution models [7], to reveal the historical spread of a fungal pathogen linked to declines of amphibians [8] and to examine historical responses of butterflies to climate change [9]. The public and private institutions that manage collections cover practically all-geographic areas with increasing levels of sampling density and taxonomic coverage over the last 500 years, and together their global collections form an infrastructure that is used annually by tens of thousands of scientific visitors. The vast majority of these collections have no digital records, and are only accessible to a handful of specialists working within each institution. As a consequence these collections remains largely unknown to the majority of potential users, with access limited by the number of visitors that each institution can host.

The sheer scale of natural history collections requires an unprecedented digitization effort to make these scientific specimens more widely accessible [10,11] and many national digitization activities are underway, such as the Digital Collections Programme at the Natural History Museum in the United Kingdom (henceforth, NHM), which holds over 80 million specimens and has a target of digitizing 20 million of these within the next five years. Similar initiatives have been put in place by the National Science Foundation, USA (Integrated Digitized Biocollections, iDigBio; https://www.idigbio.org/), the Naturalis Biodiveristy Center in Holland (at least 37 million objects by mid-2015; https://science.naturalis.nl) and the Atlas of Living Australia (http://www.ala.org.au/).

Advances in digital imaging technology are central to these digitization efforts, yet the collection of these images represents just one element of the digitization task [12]. The compilation of metadata from the billions of labels associated with these specimens, coupled with the task of persistently linking the images and metadata to the physical specimens and the publications in which they are described, represents a much greater challenge. Few collections can be more challenging than those of pinned insects—the NHM alone has more than 33 million pinned insect specimens, constituting more than 40% of the museum’s entire collection. It is neither practical nor cost-effective to digitize so many specimens individually. As a result, several whole-drawer scanning technologies have been developed [13,14] that reduce the imaging task by several orders of magnitude. This approach can be applied to digitize other collections objects such as microscope slides, 3D dry-preserved specimens (e.g., fruits, lichens and fungi) and fossils. Drawer-level digitization has become the most practical way of unlocking the research potential for natural history collections. For example, at the NHM a single scanning instrument (described further in Materials and Methods) can produce up to 70 high-resolution drawer images per day. Files are between 100 and 800 megabytes (MB) in size and each can contain images of well over a thousand individual specimens. Whole-drawer images are useful in their own right, either for collections audits or for remote identification. However specimen-level digitization, i.e., creation and association of specimen metadata with images of individual specimens, remains a laborious and largely manual process. Automatic segmentation of multi-specimen images would remove a major bottleneck in the digitization of natural history collections, by significantly reducing the time required for imaging and record creation.

General-purpose image-processing tools such as the GNU Image Manipulation Program (GIMP; http://www.gimp.org/) and ImageJ (http://imagej.nih.gov/ij/) have been proposed for the task for automatic segmentation of images [15,16] but such software is not optimised for processing the volume of large image files that are produced by mass-digitization programmes. Blagoderov et al. [11] presented a prototype for segmentation and data capture—Metadata Creator—that allowed images of individual specimens to be cropped from multi-specimen images but this software requires that the user manually draw a bounding box around each specimen. This laborious process makes it unsuitable for mass-digitization activities. Similar solutions exist for related activities within the Atlas of Living Australia (ALA) project and GigaPan service, but in both cases require manual drawing of rectangles to select subimages and do not allow for metadata association beyond simple text comments (summarised in Table 1).

**Table 1:** Comparison of features in *Inselect* with current multi-specimen image segmentation solutions.

Solution	Format	Segmentation Algorithm	Metadata capture	Barcode recognition	Modularity	Open source
ALA	Online	Manual	Simple text	No	No	Yes
Gigapan	Online	Manual	Simple text	No	No	No
ImageJ	Desktop	Automated + Manual	No	No	Yes	Yes
GIMP	Desktop	Automated + Manual	No	No	Yes: scripts	Yes
*Inselect*	Desktop	Automated + Manual	Structured fields, flexible template lookups, verification	Yes: 2D, 1D	Yes: plugin support	Yes

The lack of software to support efficient post-processing workflows associated with whole-drawer scanning has hampered the take up of mass-digitization activities [11,12]. Such tasks include but are not limited to:

- automated segmentation (the detection and placement of a bounding box around each specimen within multi-specimen images);
- automated detection and reading of one- or two-dimensional (matrix) barcodes;
- manual refinement of bounding boxes;
- association of specimen images with corresponding metadata (the addition and editing of drawer-, bulk-, and specimen-level metadata such as catalog number, taxonomic group, geographical data and physical location etc.);
- transcription of label data through manual or automated (optical character recognition) processing; export of metadata to structured files of common formats;
- saving individual cropped specimen images at the full available resolution and;
- preserving the associations between cropped images of specimens and specimen metadata (e.g., for import into collections management software).

### *Inselect*

We present *Inselect*—a modular, easy-to-use, cross-platform suite of open-source software tools designed to address the image processing needs of large-scale digitization projects. The desktop application implements automatic image segmentation, manual editing of bounding boxes, automated barcode recognition, and association of metadata with images of individual specimens. The most important and time-consuming functions are also accessible through command-line tools that operate on batches of images without human intervention, for example being run as overnight processes. Our goal was to make it straightforward to integrate *Inselect* into existing mass-digitization workflows, such as those operated by major digitization programmes.

The software is written in Python (programming language, http://www.python.org/), NumPy (Python scientific computing package, http://www.numpy.org/), OpenCV (computer vision library, http://opencv.org/) and QT (application development framework, http://qt-project.org/). All packages are mature, portable, open-source software projects with active user communities, providing a degree of assurance that the project will remain sustainable. The software runs on the three major desktop operating systems—Windows, Mac OS X and Linux. Source code, installers and open issues are at https://github.com/NaturalHistoryMuseum/inselect/. We describe *Inselect*, assess its performance and shortcomings, and make recommendations for future developments.

### Desktop application

**Fig 1:**
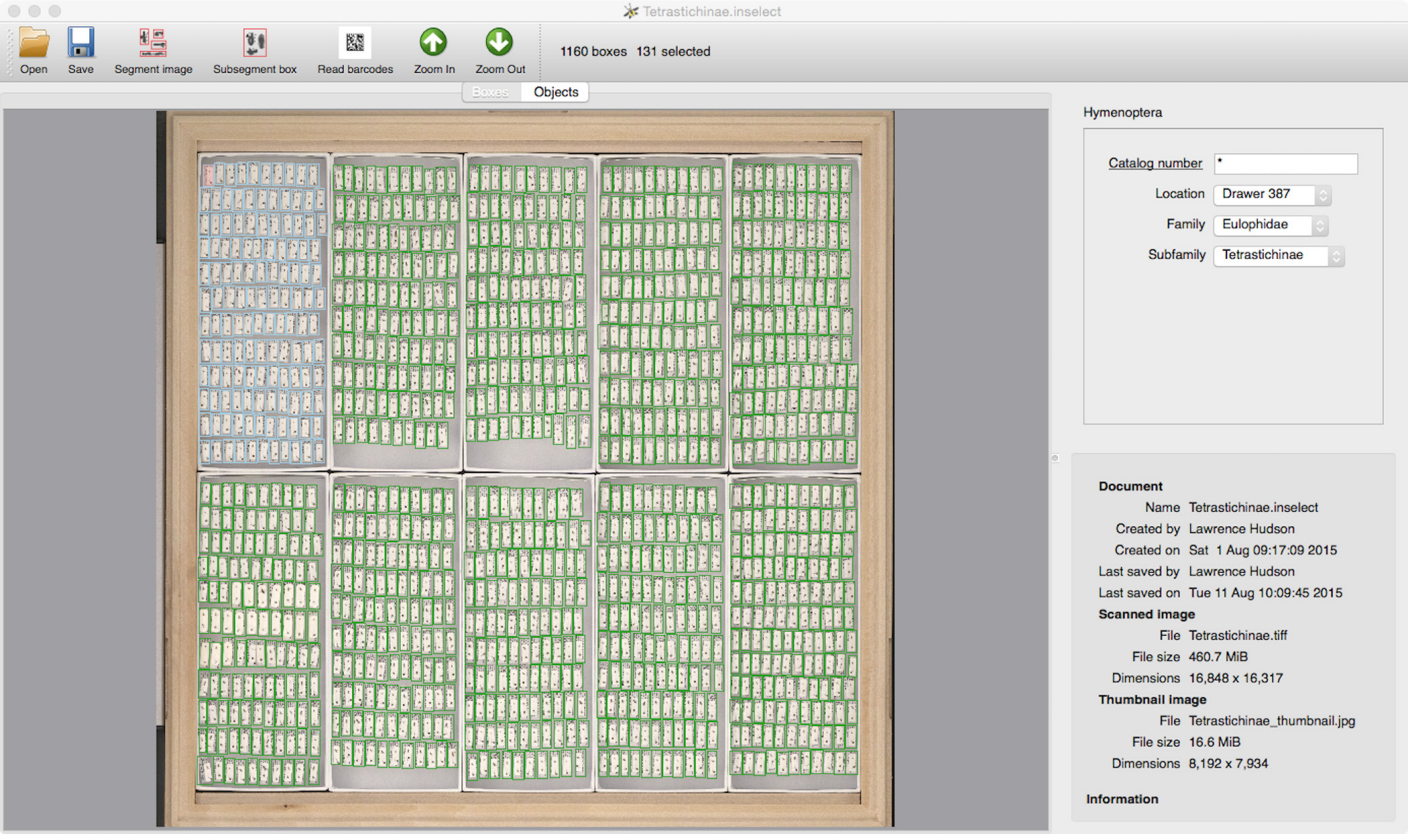
The ‘Boxes’ view. This view shows the scanned image together with bounding boxes around specimens. This document is a scan of 1,160 individuals of Tetrastichinae (a subfamily of wasps). The boxes for one tray of specimens are selected, shown outlined in light blue.

An *Inselect* document is made up of original full-resolution scanned image (all commonly encountered file formats are supported), a lower-resolution Joint Photographic Experts Group (JPEG) thumbnail (customizable dimensions, default of 4,096 pixels in width) and a list of bounding boxes together with their associated metadata. *Inselect* presents two views of these data, each designed with different tasks in mind. The ‘Boxes’ view (Fig 1) shows the complete image together with the bounding box around each individual specimen. The ‘Segment’ commands runs an automatic segmentation algorithm, which detects individual specimens and replaces existing bounding boxes. The user can then create, delete, move and resize boxes using the mouse and/or keyboard, making it a simple task to refine the results of the segmentation process. The panel on the right contains metadata fields.

The user has complete control over the list of fields and any associated validation. In the Edit menu, ‘Choose template’ allows the user to select an ‘.inselect_template’ file that contains metadata fields definitions. Templates are written in YAML (YAML Ain’t a Markup Language -http://yaml.org)—a structured text format that is easy to learn and that can be edited using a plain-text editor. Fig 1 shows a template called ‘Hymenoptera’ with one numeric field (‘Catalog number’, which can be populated by values of object barcodes–see below) and three fields with drop-down lists of values. The metadata fields reflect the currently selected boxes, making it easy to enter metadata for a single specimen, a group of specimens, or to all the specimens in the initial image (e.g., a taxon name or geographic location). The 131 selected boxes (Fig 1) have the same values for ‘Location’, ‘Family’ and ‘Subfamily’ but different values of ‘Catalog number’. The template specifies that each of these four fields is mandatory. Any boxes that fail validation (e.g., missing mandatory values) are shown with a red background—the first of the 131 selected boxes in Fig 1 is shown in red because it lacks a value of ‘Catalog number’. *Inselect* templates permit comprehensive field validation such as ‘an integer value greater than zero’, ‘a latitude’, ‘a longitude’ and ‘a date in the form YYYY-MM-DD’. For more complex cases, field validation can be given as a regular expression. For example, the NHM templates use the regular expression '^[0–9]{9}$'—exactly nine digits with no letters, no punctuation and no leading or trailing whitespace–for the ‘Catalog number’ field. The user can specify other properties in the template, such as the width of the low-resolution thumbnail image (default of 4,096 pixels). A complete description of the format, along with example templates that are used for NHM’s digitization projects, are available in the github repository: https://github.com/NaturalHistoryMuseum/inselect-templates. The built-in 'Simple Darwin Core terms' template, which contains all Simple Darwin Core terms (http://rs.tdwg.org/dwc/terms/simple/; [17]), can be used by selecting the ‘Default template‘ command under the Edit menu, Metadata can be exported to comma-separated values (CSV) files and included in the file name of segmented images.

**Fig 2:**
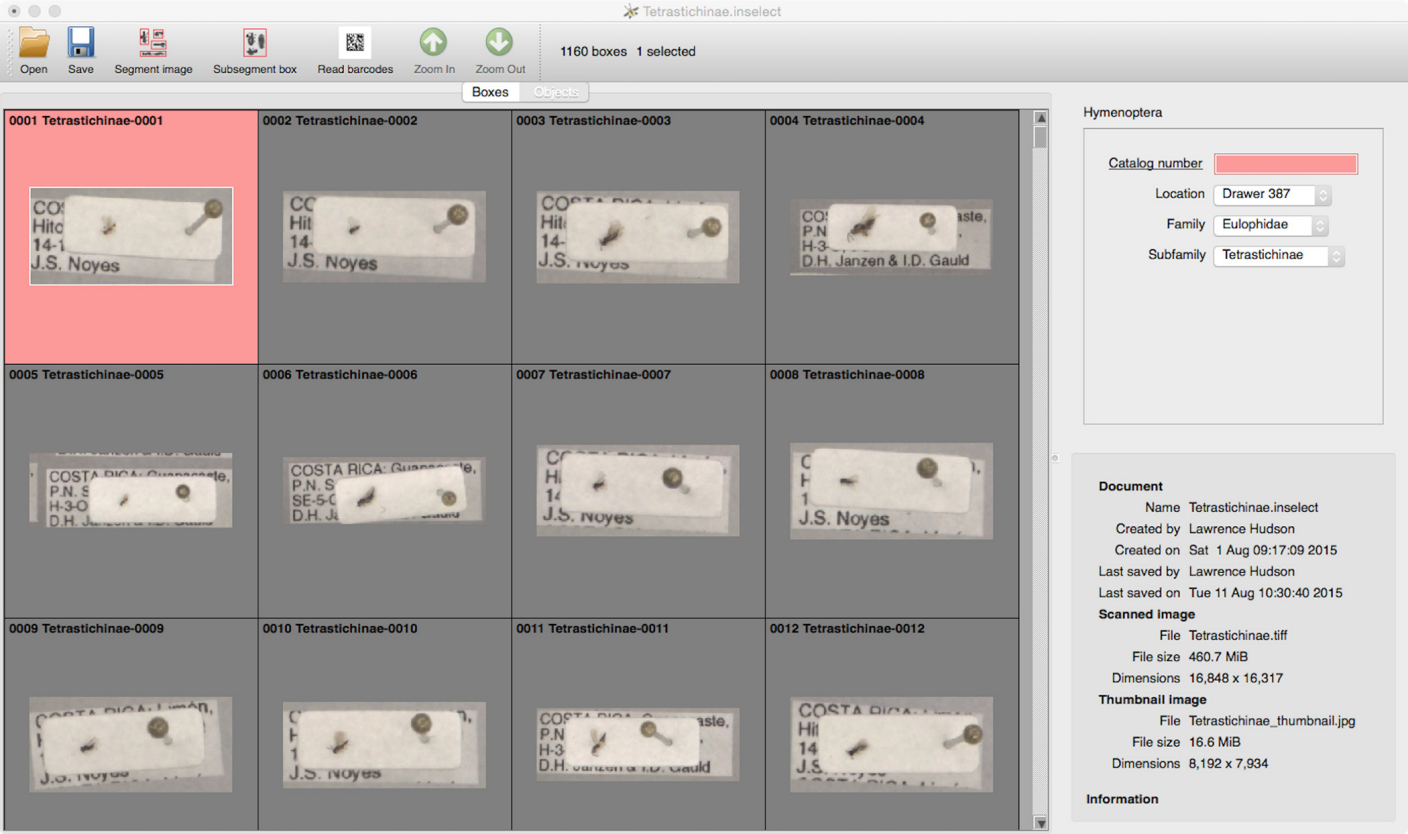
The ‘Objects’ view. This view shows objects in a grid. The selected object lacks a mandatory metadata field so is shown in red.

The ‘Objects’ view (Fig 2) shows individual images either in a grid or with a single image expanded. The first box lacks a value of ‘Catalog number’ and so is shown with a red background. The user can rotate images individually or in groups, making it easy to transcribe label information into metadata fields. Rotation is also applied to the cropped object images, when these are saved.

*Inselect* displays the low-resolution thumbnail image, which is small in size, quick to read and takes up relatively little space in-memory; the full-resolution file (which might be many hundreds of megabytes in size) is loaded only as required, for example when saving the individual cropped specimen images.

The desktop application supports plugins—code modules that are able to examine and possibly modify the list of bounding boxes and their associated metadata. Plugins can access the low-resolution thumbnail image and, if necessary, the full-resolution scanned image. The software currently has plugins for automated segmentation of the entire image and for sub-segmentation of a single bounding box (see ‘Segmentation algorithms’ below).

Many institutions use barcodes to uniquely identify specimens. *Inselect* therefore provides a ‘Read barcodes’ plugin, which reads the values of any barcode(s) within each box and places value(s) in the ‘Catalog number’ metadata field. Barcodes typically take up just a small fraction of the area of an image (e.g., S1 Fig); they can be smudged or damaged and can be placed at an angle, making it a non-trivial task to quickly and reliably detect and decode barcodes. *Inselect* includes two open-source libraries: zbar (http://zbar.sourceforge.net/), which reads one-dimensional barcodes and QR codes; and libdmtx (http://www.libdmtx.org/), which reads Data Matrix barcodes. We found that commercial libraries were faster and more reliable than the two open-source decoders. *Inselect’s* ‘Read barcodes’ plugin therefore also supports the best performing of the commercial libraries—Inlite Clearimage (purchase or download for evaluation from http://www.inliteresearch.com/barcode-recognition/). The user can select which of these libraries to use by selecting the “Configure ‘Read Barcodes’” command, under the Edit menu.

### Command-line tools

Each command-line tool makes available some of the functionality of the desktop application in a form that is convenient for unattended processing of images in batches:

- ingest: reads each scanned image creates and saves an empty *Inselect* document, along with a thumbnail image;
- segment: runs the segmentation algorithm for each *Inselect* document that does not already contain bounding boxes;
- save_crops: for each *Inselect* document, writes specimen images cropped from the high-resolution image; and
- export_metadata: for each *Inselect* document, writes a CSV file containing metadata.

**Fig 3:**
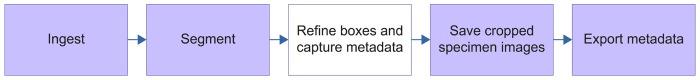
Typical workflow. All processes are offered by the desktop application. Shaded boxes indicate processes that can be performed by the command-line tools, which can work on batches of images and documents.

Each of these tools corresponds to a shaded box in the typical *Inselect* workflow shown in Fig 3.

### Segmentation algorithms

Both of *Inselect*’s algorithms operate on thumbnail images. The automatic segmentation algorithm converts the image to the CIELAB (*Commission internationale de l'éclairage*, L*a*b) colour space and then adds Gaussian blur in order to remove noise. It then applies Sobel filters in x and y directions and applies a threshold, resulting in a binary image (i.e., pixels are either ‘off’ or ‘on’) where ‘on’ indicates that an edge in the source image. The algorithm then detects contours around each edge and computes the bounding box around each contour. Contours are processed recursively in order to detect edges-within-edges, such as specimens within insect trays. The result of the algorithm is a list of bounding boxes.

The sub-segmentation algorithm is applied by the user to a single bounding box that contains many specimens—a situation that can arise when the automatic segmentation algorithm was unable to discriminate between specimens. The user marks each individual specimen within a box using shift+left mouse click. The sub-segmentation algorithm applies a watershed technique, in which the image is considered to be a topographical surface with peaks and valleys: each ‘valley’ (indicated by a user-designated marker) is ‘filled’ with a different colour ‘water’ until all ‘peaks’ are submerged. The resulting ‘lakes’ of different colours indicate the extent of each specimen. The result of the algorithm is a list of bounding boxes.

Based on an initial period of exploration with a variety of images from the NHM’s collection, all free parameters of both algorithms were hard-coded within the *Inselect* software.

## Materials and Methods

### Test images

We evaluated the performance of the software using 804 multi-specimen Tagged Image File Format (TIFF) images of specimens from the NHM’s collections. Images were captured using the SmartDrive SatScan (http://www.smartdrive.co.uk/) collection scanner, which is capable of producing high-resolution images of entire collection drawers. A camera (UEye-SE USB CMOS model UI-1480SE-C-HQ, 2560×1920 resolution) and an attached lens (Edmund Optics telecentric TML lenses model #58428 0.3× or model #56675 0.16×) is moved in two dimensions along precision-engineered rails positioned above the objects that are to be imaged. A combination of hardware and software provides automated capture of high-resolution images of small regions of interest, which are then assembled (“stitched”) into a single panoramic image by proprietary software (Analyse, by SmartDrive). This method maximizes depth of field of the captured images and minimizes distortion and parallax artefacts.

We used scanned images of pinned insects stored in collection drawers of between 400 x 500 mm and 555 x 572 mm in size, with or without unit trays. Some of the scanned images contain, in areas where no specimens are present, paper with a printed Penrose tiles pattern–these were added in order to aid earlier versions of the stitching algorithm. We also tested *Inselect* using scans of standard-size microscope slides, laid out for imaging in a rectangular grid containing 72 sockets arranged in six columns and twelve rows, and large-sized microscope slides, arranged in a grid of six columns and eight rows; some sockets were empty in some scans. We make the thumbnail images (on which the segmentation algorithm operates) of our complete test dataset available at http://dx.doi.org/10.5519/0018537.

### Performance

For each image, we computed or measured:

- the dimensions of the scanned TIFF image (in pixels);
- the size of the scanned image file (in MB);
- the time to ingest (i.e., read the scanned image, save a JPEG thumbnail image of 4,096 pixels in width, and create an empty *Inselect* document);
- the size of the thumbnail image file (in MB);
- the time to segment and
- the number of boxes found by segmentation.

We picked 30 images at random and manually refined the bounding boxes that were detected by the segmentation algorithm. This involved correcting false positives (removing boxes where there was no specimen), false negatives (creating boxes where specimens did not have one) and adjusting the size of boxes that did not encompass the entire specimen and associated labels. We recorded the time taken to refine the bounding boxes and the actual number of specimens on the image.

All tests were carried out on an eight-CPU Dell Precision T3610 workstation with 32GB RAM, running Ubuntu Linux 14.04 (http://www.ubuntu.com/), Python 2.7.9, NumPy 1.9.1, OpenCV 2.4.9 and QT 4.8.6. The version-control tags for these two experiments are ‘performance-experiment-1’ and ‘experiment-1-refinement’ respectively.

## Results

**Fig 4:**
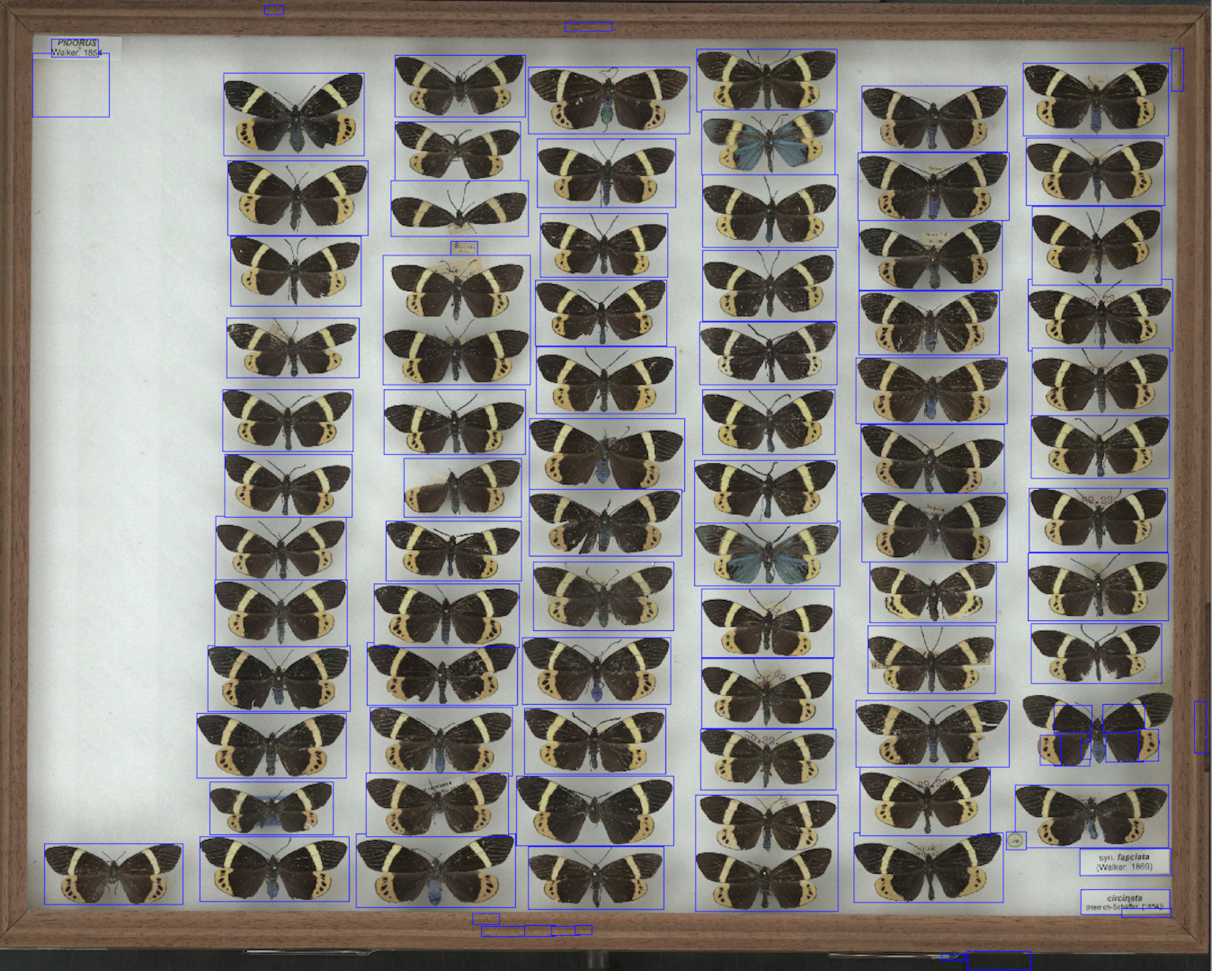
Segmented image of moth specimens. Example of a segmented image of Chalcosiinae (a subfamily of moths) specimens.

**Fig 5:**
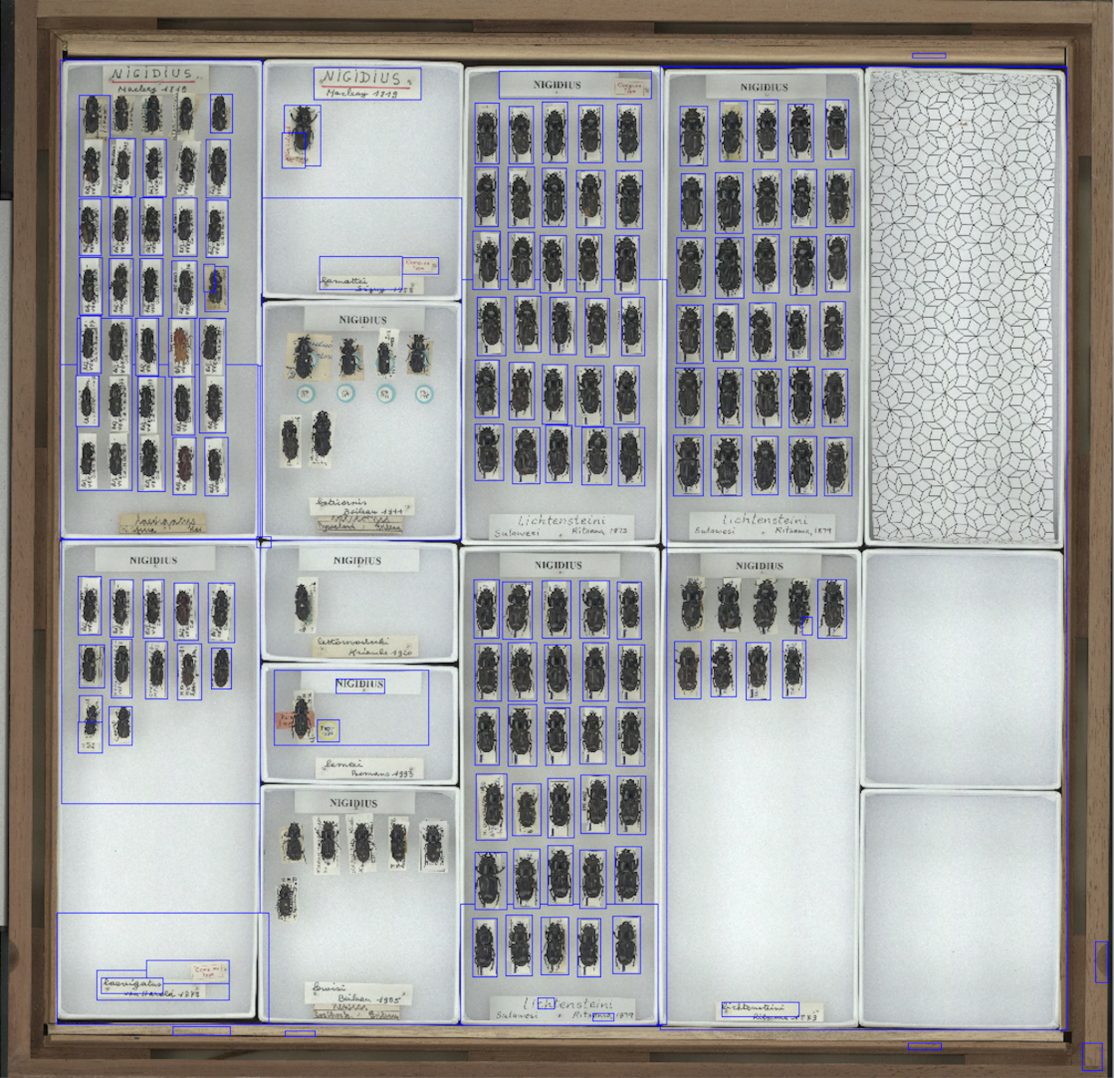
Segmented image of beetle specimens. Example of a segmented image of Lucanidae (stag beetles) specimens.

**Fig 6:**
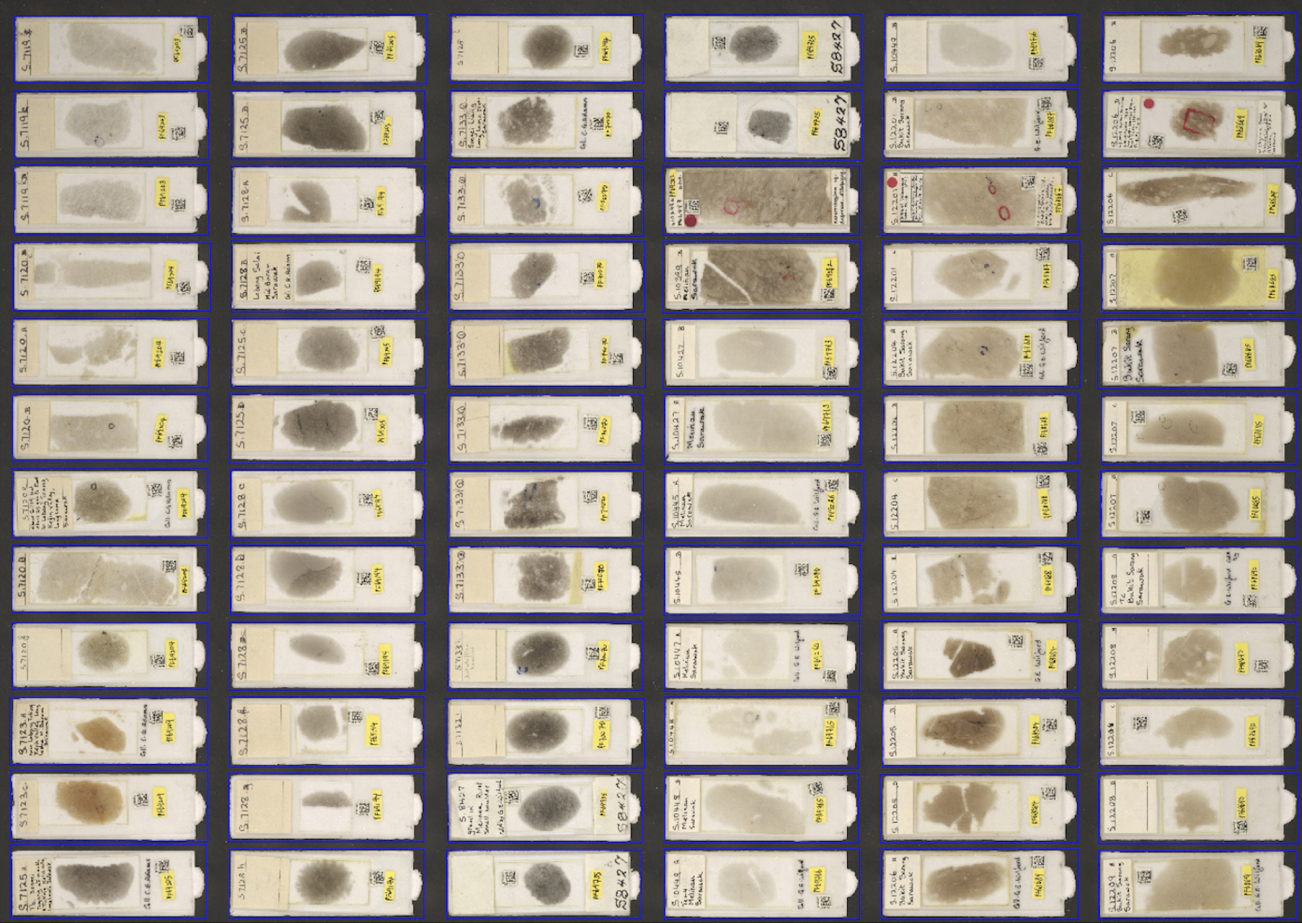
Segmented image of microscope slides. Example of a segmented image of microscope slides of benthic Foraminifera in a rock thin section.

The mean dimensions of the scanned images were 18,131 x 15,268 pixels. The complete set of scanned images took up 341GB on disk; file sizes varied between 111MB and 796MB, median 429MB (Table 2). Examples of segmented images are shown in Figs 4–6. Median thumbnail images were nearly two orders of magnitude smaller than the full-resolution scanned images (S2 Fig). The whole experiment took 2 hours, 29 minutes to run. Ingestion times are shown in S3 Fig. Segmentation time is not explained by the number of bounding boxes detected (S4 Fig). We refined the bounding boxes of 30 images picked at random. The median time to refine was 108.5 s (minimum time 8.5s, maximum time 413.0s; S5 Fig) and the number of bounding boxes detected by segmentation was not a good predictor of the actual number of specimens (S6 Fig).

**Table 2:** Scanned images by group.

Group	Description	N images	File sizes (MB)		
			Min	Median	Max
Brahmaeidae	A family of moths	30	721.1	734.5	796.0
Cerambycidae	The family of longhorn beetles	2	110.9	115.5	120.1
Chalcidoidea	A superfamily of wasps	2	419.9	423.2	426.6
Chalcosiinae	A subfamily of moths	271	396.0	431.0	469.0
Charaxinae	A subfamily of butterflies	67	368.3	428.3	504.3
Coccinellidae	Ladybirds	7	401.5	414.0	415.2
Embioptera	Webspinners	2	429.5	430.8	432.1
Limacodidae	A family of moths	4	410.5	417.0	423.7
Lucanidae	The family of stag beetles	240	214.2	435.4	471.9
Lycaenidae	A large family of butterflies	53	406.8	426.8	436.9
Microscope slides	Benthic Foraminifera in a rock thin section	105	378.1	404.5	433.2
Microscope slides (large)	Benthic Foraminifera in a rock thin section	13	378.3	387.8	408.4
Mixed moths and butterflies	Mixed moths and butterflies collected in Madagascar	2	440.5	613.4	786.4
Mycalesina	A group of satyrid butterflies	4	400.4	429.5	455.3
Neuroptera	Lacewings and their relatives	2	430.1	433.7	437.3

## Discussion

### Ingestion and segmentation performance

**Fig 7:**
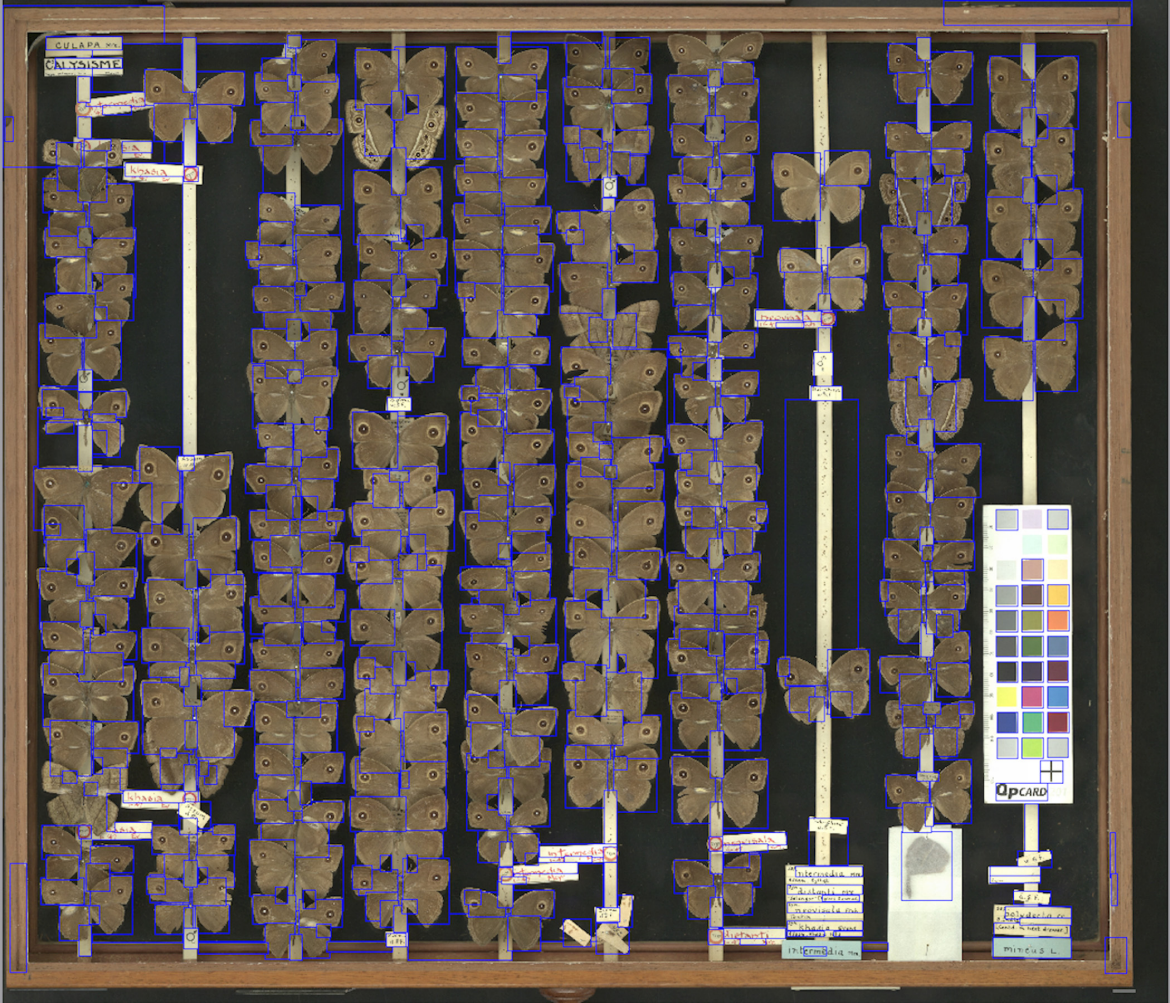
Segmented image containing overlapping specimens. Example of segmented image of Mycalesina (a group of butterflies).

It took just 2 ½ hours to ingest and segment just over 800 images, which represents more than twice the weekly output of a SatScan machine running at full capacity. Some images contained overlapping specimens (e.g., Fig 7). Not only are such images challenging for any segmentation algorithm but the resulting cropped specimen images are of questionable use; arguably, these drawers should be re-curated and re-imaged. As might be expected given the way that JPEG compression works, thumbnail size is a function of image complexity rather than size of the full-resolution scanned image (S2 Fig). We expected time to ingest (read full-resolution TIFF, resize in memory and write JPEG thumbnail) to scale linearly with the size of the full-resolution image file. Variation in ingestion time (S3 Fig) could be due to blocked PC resources (CPU, RAM, hard-disk) but the PC used to carry out the tests has a high specification, and the image files were on their own physical hard disk that was not being used for other tasks. JPEG compression speed is more likely to explain the variation given that this is correlated with image complexity, and that this complexity is highly variable across each drawer.

### Application in natural history collection digitization workflows

Nelson et al. [12] described three dominant digitization workflows for natural history collections: (1) data capture with occasional specimen imaging, (2) parallel data and specimen image capture, and (3) imaging of specimens and labels followed by data capture from the image. We consider the third workflow as the most efficient process for mass digitization of very large collections. This allows operators to perform simultaneous image and data capture for multiple specimens, thus significantly increasing throughput (S7 Fig).

*Inselect* was developed primarily to suit the needs of the mass digitization program within the Natural History Museum, and can, we hope, also be used by the many organisations with collections that share the following characteristics:

- extremely large size;
- reasonably complete taxonomic index (list of taxa represented in the collection);
- complete record of collection lots (i.e., multi-specimen and mixed taxon collections) and
- very low percentage of specimen-level records.

Under these circumstances one of the most pressing priorities is a”broad-and-thin” approach to digitization: the collection of essential specimen-level data allowing complete collection audit and providing a specimen level platform to add metadata in future. At a minimum this includes the specimen's determination (i.e., taxon name) and its physical location within the collection. *Inselect* has proven to be a very useful tool for the most challenging parts of the NHM’s collections, such as pinned insects, and it can be easily applied to other areas, for example, environmental studies and quantitative analysis of trap samples (e.g., “invertebrate soups” or sticky traps, see S8 Fig).

In the course of the NHM Slide Digitization Pilot project, which will digitize 100,000 microscope slides in eight months, *Inselect* received extensive user acceptance testing. Results to date show that throughput is as high as 5,000 slides per day, per person, for processing multi-slide images through *Inselect*. This includes tasks associated with image segmentation and refinement, barcode recognition and association with minimal metadata (taxon name and physical location in the collection). Upon completion of the project, the entire set of time and motion studies alongside the associated workflow will be described in a separate publication. Drawers of curated pinned insects, as a rule, do not require additional preparation; therefore, the imaging output can be up to 70 SatScan images per day, resulting in 3,500–70,000 specimens per day available for *Inselect*.

### Future developments

#### Segmentation algorithms

The high throughput of mass-digitization activities makes it important to minimize the amount of manual intervention required. The NHM’s SatScan instrument can generate up to 70 multi-specimen images per day. The median user-time required to refine segmented images of 109 s (S5 Fig) means that 70 *x* 109 */* 3600 *=* 2.1 person-hours could be required to refine bounding boxes for a day’s worth of images from a SatScan machine. In the worst case (413 s), this refinement time increases to more than eight hours. Therefore segmentation algorithms should be as accurate as possible and we suggest that there is a need for a formal method (and supporting software) that allows segmentation methods and their associated parameter sets to be scored and ranked. Such a score should consider performance, false positives and false negatives. The outputs of such an activity might be a library of algorithms and/or parameter sets, geared towards different specimen types. The dataset of 804 images used in the present work (available at http://dx.doi.org/10.5519/0018537) constitutes a benchmark dataset against which segmentation algorithms can be measured. *Inselect*’s modular architecture and its provision of plugins make it a suitable platform for such an investigation.

#### Desktop application

The desktop application lacks some of the polish that is expected of modern software such as ‘undo’ and localization. Other desirable features include the ability to filter and order bounding boxes by size and/or area in order to aid refinement, support for Exchangeable Image File Format (EXIF) tags and integration with industry-standard image processing tools such as Adobe Photoshop (http://www.adobe.com/products/photoshop.html). The plugin architecture makes a possible range of developments, such as additional segmentation algorithms and optical character recognition of label text within bounding boxes.

*Inselect* has been tested using specimens from the NHM’s entomological and micropalaeontological collections, as well as a limited number of specimens from Continental European collections. We would like to test the software against a greater diversity of museum specimens and institutions to ensure that it can accommodate variation in the storage and mounting of these specimens.

Despite these limitations, *Inselect* represents a substantial contribution to the tools available to support mass-digitization of natural history collections. The desktop application and its associated command-line tools have been designed to efficiently handle the high numbers of large image files produced by mass-digitization activities. The combination of a modular architecture, desktop application and scriptable technology makes it a relatively simple task to integrate *Inselect* into existing and workflows. Bug reports, feature requests and ideas can be viewed and created at https://github.com/NaturalHistoryMuseum/inselect/issues. We are actively developing *Inselect* and we greatly value all comments and suggestions.

## Supporting Information

S1 Fig A cropped specimen image containing a barcode. A scan of a microscope slide that contains a Data Matrix barcode.
(TIFF)

S2 Fig Thumbnail file size against file size. Numbers in brackets in the legend are the number of files in that group.
(EPS)

S3 Fig Ingestion time against file size. Ingestion time is the time taken to read the scanned image and save a JPEG thumbnail image of 4,096 pixels in width. Numbers in brackets in the legend are the number of files in that group.
(EPS)

S4 Fig Segmentation time against number of boxes found. Numbers in brackets in the legend are the number of files in that group.
(EPS)

S5 Fig Distribution of the time to refine 30 segmented images. For 30 images picked at random.
(EPS)

S6 Fig The actual number of specimens against the number of boxes detected by segmentation. For 30 images picked at random.
(EPS)

S7 Fig Imaging digitization workflow. a. Original single-specimen imaging and data capture (after Nelson 2012, modified); b. multi-specimen imaging and data capture.
(EPS)

S8 Fig Alternative use of *Inselect* for trap sample. Count estimation (1,064 specimens) using *Inselect* on an image of a yellow sticky trap used in an environmental assessment study.
(TIFF)
