# Supplementary figures and images for: Inselect: Automating the Digitization of Natural History Collections
Source: PLoS One. 2015 Nov 23;10(11):e0143402. doi: 10.1371/journal.pone.0143402 (PMC4658125; doi:10.1371/journal.pone.0143402)

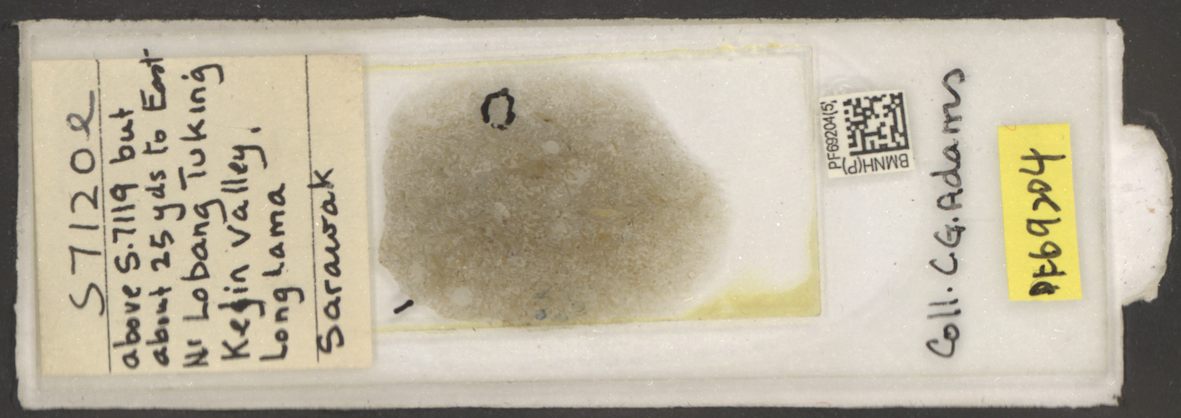

Supplement: S1 Fig — A scan of a microscope slide that contains a Data Matrix barcode. (TIFF) [file pone.0143402.s001.tiff]

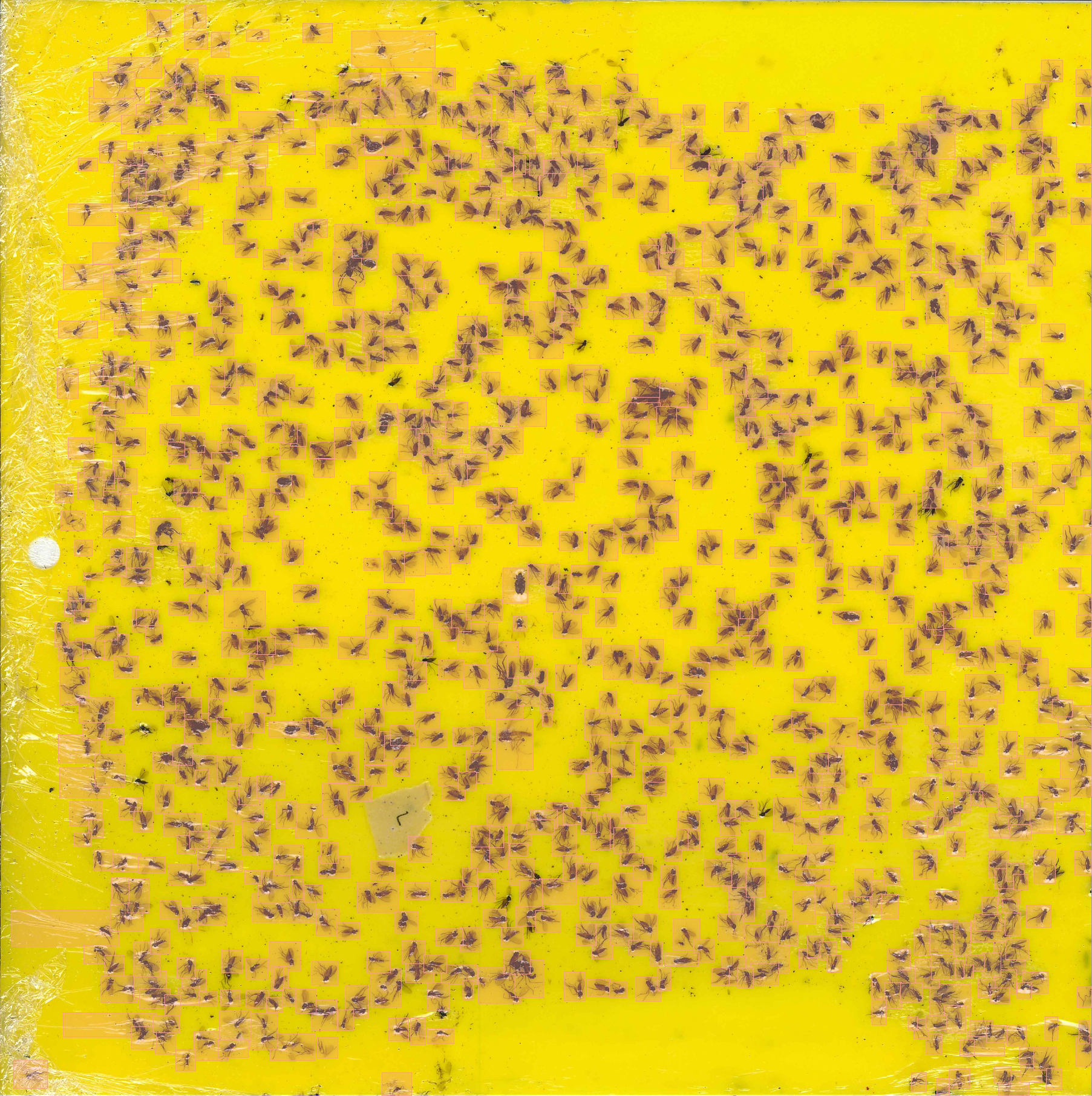

Supplement: S8 Fig — Count estimation (1,064 specimens) using Inselect on an image of a yellow sticky trap used in an environmental assessment study. (TIFF) [file pone.0143402.s008.tiff]
